# Understanding the relationship between built environment features and physical activity in the Caribbean: A scoping review

**DOI:** 10.1016/j.dialog.2022.100088

**Published:** 2022-12-05

**Authors:** Kern Rocke, Christina Howitt, Ian Hambleton

**Affiliations:** a The George Alleyne Chronic Disease Research Centre, Caribbean Health Research Institute, The University of the West Indies, Barbados; b Faculty of Medical Sciences, The University of the West Indies, Cave Hill Campus, Barbados

**Keywords:** Built environment, Physical activity, Caribbean, Small Island developing states, Latin America

## Abstract

**Background:**

Transforming the urban infrastructure to become safe, inclusive and sustainable remains a challenge in most developing settings. The Caribbean has high burdens of physical inactivity and non-communicable diseases. Therefore, understanding the role of the built environment (BE) in modifying individual activity is important for informing the design of community interventions to improve levels of physical activity (PA). Anecdotally, there is limited evidence on the BE in the Caribbean, however evidence from other Small Island Developing States (SIDS) and from Latin America (LA) may offer useful information given their similar urbanization profiles and shared geo-collaborative histories.

**Objective:**

Our review identifies and characterizes individual features of the BE and examines their relationships with PA outcomes.

**Methods:**

We systematically searched a range of multi-discipline research databases, including studies from SIDS and LA that objectively measured BE features as an exposure and PA as an outcome between 2010 and 2021. Grey literature was not considered for this review. We characterized BE measures into 9 neighbourhood design domains using the Walkability for Health framework, and mapped gaps in the published evidence. We performed a narrative summary of BE-PA relationships, focusing on association strength and direction of effect.

**Results:**

Fifty-one studies from published scientific literature in Brazil, Colombia, Mexico, Chile, and Singapore were included that described 306 BE-PA relationships. The BE was mostly characterized by number of and proximity to spaces for social interaction, green spaces, increasing housing density or street connectivity, and mixed residential and commercial land use. BE-PA relationships, although inconsistent, largely promoted PA.

**Conclusion:**

Although the review is suggestive of the benefits of the benefits of BE interventions for promoting active commuting and leisurely PA, translational research is needed to understand whether results can be successfully adapted for SIDS, which often have an urban structure defined by a single urban centre with connections to outlying communities.

## Highlights

• Peer-reviewed evidence in the Caribbean on objectively-measured built environment features and physical activity is lacking.
• Assessment of built environment evidence from SIDS & LA focused mostly on greensapce, street connectivity & mixed land use.
• Recognised built environment enablers of physical activity should consider local sensitivities when applied to the Caribbean.

## 1 Introduction

Physical activity (PA) of any type or intensity has the potential to reduce morbidity and mortality from non-communicable diseases in children and adults [1]. Despite the well-known benefits of an active lifestyle, levels of physical inactivity continue to increase globally, particularly among economically disadvantaged communities and developing economies [2,3]. Reducing this burden has been identified as a public health priority by the World Health Organization [4]. Studies from diverse populations have suggested that the built environment (BE) might be a powerful determinant of physical activity, including decisions related to the engagement in outdoor PA [[5], [6], [7], [8]].

Most evidence examining associations between BE features and PA comes from high-income economies [9,10]. Evidence from developing countries, though limited, has been reviewed by Day and colleagues. Studies in these settings more often consider rural as well as urban environments, recognizing the differing levels of urbanization seen across the development continuum [11]. Most evidence suggests that physical activity can be promoted by integrating where people live with regular destinations such as shops, parks, and offices [11,12]. Previous reviews found differences between developing and developed settings suggesting that geographical and cultural differences play an important role in how individuals interact with their built environment. Furthermore, evidence has also shown that traffic safety and increasing residential density were positively associated with PA in developing and developed settings respectively [[11], [12], [13]].

There is relatively little evidence emerging from geographically small settings, such as the Small Island Developing States of the Caribbean. Countries within this region have similarly high burdens of physical inactivity, overweight and obesity, poor diet and engagement in risky health behaviours such as: smoking and alcohol consumption [14,15]. Caribbean countries are characterized by their unusual urban structures, with often a single urban centre with connections to outlying communities, for example along a coastal perimeter. Although planned urbanization can integrate the three pillars of development – economic, environmental and social – urbanization among the Small Island Developing States (SIDS) has been rapid, with for example urbanization rates in the Pacific between 2010 and 2015 of 4.3%, in Haiti (3.9%), Trinidad and Tobago (2.2%), compared to a global average of 1.7% [16]. The unusual urban structure of SIDS, the rapidity of urbanization and the disconnect with economic wellbeing may create special challenges for developing healthy urban living environments and built environment interventions in the Caribbean.

We are unaware of recent published evidence examining the relationship between objectively measured features of the BE and physical activity in the Caribbean. To gain some understanding of the role the BE might play in influencing PA we sought to expand our scope to settings that have similarities with the Caribbean. The SIDS share many similarities, including high burdens of non-communicable disease, small land areas leading to economic and developmental challenges due to limited resources, and environmental vulnerabilities [17,18]. Countries in Latin America (LA) have a long history of regional collaboration and integration with the Caribbean primarily because of geographical proximity [19]. Therefore, in this review we have summarised the evidence base for the influence of BE characteristics on physical activity in SIDS and LA. We have characterized BE measures into nine neighbourhood design domains using the Walkability for Health framework and have mapped gaps in the published evidence. We have performed a narrative summary of BE-PA relationships, focusing on strength and direction of effect.

## 2 Methods

### 2.1 Design

This scoping review has been conducted using the framework and methods published by Arksey and O'Malley, and Peters et al. [20,21]. Our full study protocol is registered on the Open Science Framework (DOI: http://10.17605/OSF.IO/PYZQE). All findings are reported using the Preferred Reporting Items for Systematic Reviews and Meta-analyses extension for scoping reviews (PRISMA-ScR) guidelines [22] [See supplement- Table S1].

### 2.2 Search strategy

We systematically searched peer-reviewed literature using a search strategy [See supplement] adapted from previous reviews by Peters et al. [23] and Smith et al. [24] and using the following online databases: MEDLINE; SciELO; CINAHL; LILACS; OARE; TRID; TRIS; Research in Progress; IBECS and Web of Science [12,25,26]. We examined the evidence between 2010 and 2021 to capture the recent surge in research activity related to the built environment and health.

### 2.3 Study inclusion criteria

We included studies for title and abstract screening meeting all the following criteria: Observational and intervention (randomized and non-randomized) studies; studies involving individuals or communities living in SIDS or LA [See supplement- Table S2 & S3]; studies reporting one or more objectively measured feature of the built environment (for example, using desktop mapping, observational field audits, remote sensing); studies reporting on PA as a primary or secondary outcome either self-reported (obtained via questionnaires, surveys, or interviews) or objectively measured.

### 2.4 Screening strategy

After removing database duplicates, we (two authors, KR & CH) independently screened titles and abstracts of articles found by the search strategy, uploading citations into the Rayyan online bibliographic database [27]. We resolved decision discrepancies through discussion and where necessary by consultation with a third reviewer (IH). We then performed full-text screening and data abstraction (KR, CH) using a data extraction form implemented in REDCap, a secure online data collection infrastructure [28]. We recorded all decisions on articles excluded at this stage of the review.

### 2.5 Built environment

To characterize measures of the built environment we used the Walkability for Health framework, which describes nine broad neighbourhood design domains considered essential for promoting physical activity through walkability [29,30]. These categories were: surveillance, experience, parking, traffic safety, community, greenspace, density, connectivity, land use [See supplement- Table S4 for details of each domain]. There are four broad methods that can be used to measure aspects of the BE: self-report, systematic observations using for example community audits, government statistics and geographic information systems (GIS) measures [23]. To limit the bias associated with subjective methods, we limited our review to objective measures of the BE: geographical information systems and observational field audits.

### 2.6 Physical activity

Ideally, we would have limited the PA outcomes to objective measures, but this would have resulted in very few included studies (*n* = 11 studies), and we chose therefore to also include self-reported physical activity. We recorded the type of physical activity, both self-reported in the form of surveys/questionnaires and objectively measured in the form of pedometers or accelerometers. Reported physical activity outcomes examined were active commuting (cycling and walking); leisure-time PA; and MVPA (moderate to vigorous PA).

### 2.7 Data synthesis

We conducted our synthesis of the charted data in two stages. First, we characterized each included study (geographic location and year of publication) and categorized the BE features examined using the Walkability for Health framework. A single study can report multiple relationships. We identified the number of relationships between BE features and PA outcomes in each included study and used these relationships to create an evidence gap map. The gap map visualizes the frequency of reported relationships as a matrix with BE domains as rows and PA outcomes as columns [31,32]. Second, we explored the reported relationships between BE features and PA outcomes, focusing on direction and magnitude of each association. Odds ratios (ORs) were the most commonly reported effect size measure, and we transformed studies with alternative measures to ORs for consistency [33]. Studies without enough data to allow this conversion were excluded for this synthesis stage (*n* = 21 relationships). When available we used estimates pre-adjusted for confounders using multivariable regression models.

Our main goal was to identify and map the available evidence on associations between BE features and PA, and we did not formally assess the risk of bias for each study. Our focus on the directional effect of BE-PA relationships means that it is important to understand the influence of publication bias on our results, and we assessed the likelihood of publication bias in our identified studies [34]. We conducted all data management, analysis and visualizations using Stata statistical software [35]**.**

## 3 Results

**Fig. 1:**
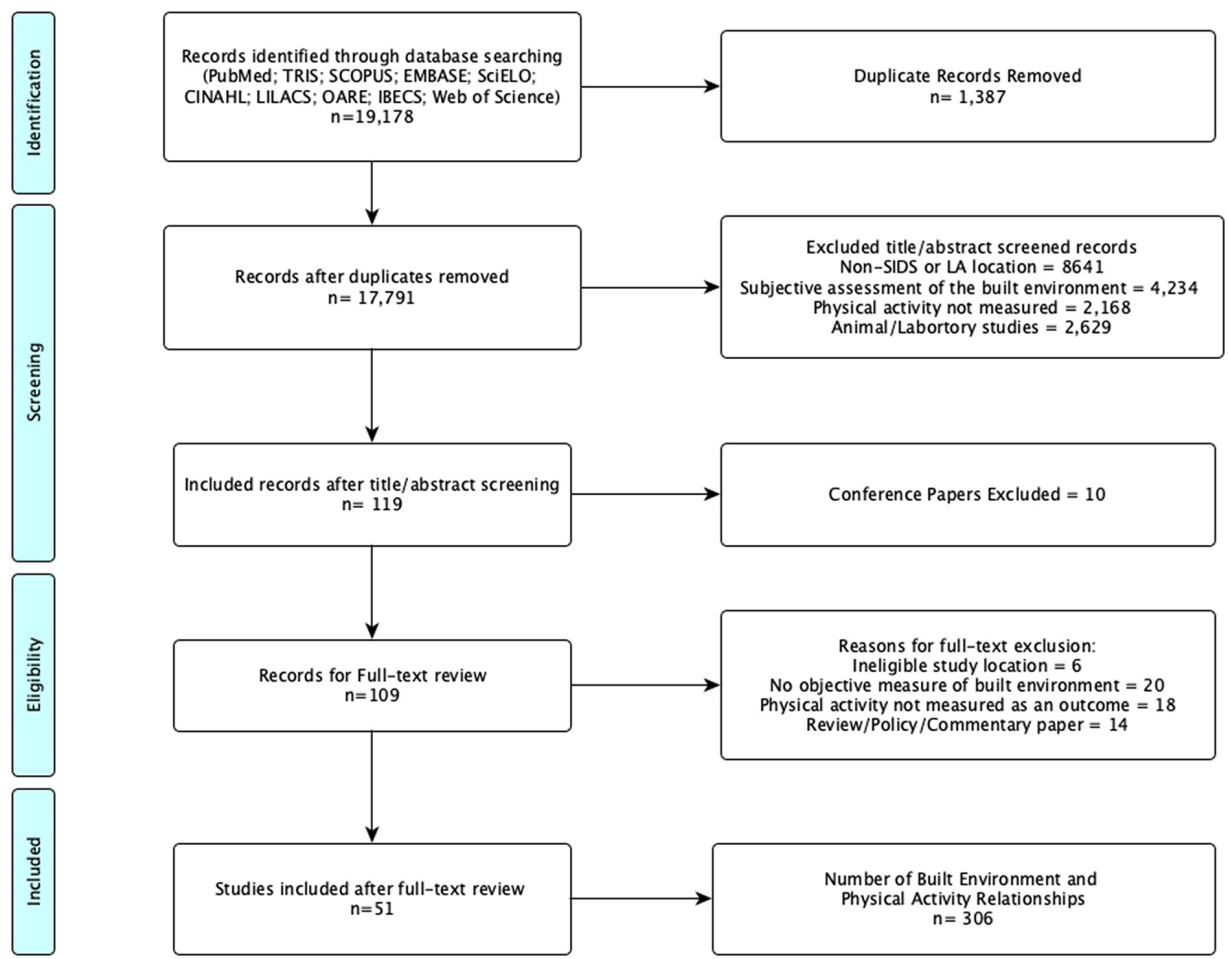
PRISMA-ScR diagram of built environment and physical activity in SIDS and Latin American countries.

Fig. 1 shows the PRISMA-ScR flow diagram of articles included and excluded for this review. Our literature search identified 19,178 potentially relevant titles/abstracts. We excluded 17,791 records for the following reasons: duplicates (*n* = 1387), non-SIDS or non-LA study location (*n* = 8641), the study used subjective assessment of the built environment either from participant perceptions or measures assessed subjectively through questionnaires and interviews (*n* = 4234), the study did not include a measure of physical activity (*n* = 2168), the study used animals or laboratory methods (*n* = 2629). We identified 119 articles for full-text review, of which 10 articles were conference abstracts with inadequate levels of published information. This left 109 records for full text review, from which a further 58 records were excluded for the following reasons: ineligible study location (*n* = 6), no objective measure of the BE (*n* = 20), physical activity was not measured as an outcome (*n* = 18), and studies were either reviews/policy/commentary papers with inadequate BE and PA information (*n* = 14). After we completed full text review, 51 articles were included for this review.

### 3.1 Study characteristics

A summary of the key characteristics of studies included are presented in Table 1. (Further details can be found in Supplement Table S5). Most included studies were cross-sectional (*n* = 50), with just one study collecting multiple data points over time [36]. Two studies used neighbourhood as their primary unit of analysis, with all others summarizing data from individual participants [37,38]. Most studies (*n* = 43) were conducted in Latin America in the following settings: Brazil, Mexico, Colombia, and Chile, with eight studies conducted in Singapore [[39], [40], [41],^38^,[42], [43], [44], [45]]. All studies were conducted in exclusively urban settings (characterized by communities/areas of high population density and presence of infrastructure for the BE). Studies reporting data from individuals included adolescent participants and older (age range across all studies: 6 to 98 years). Studies mainly adjusted for socio-demographic confounders in their analyses (such as age, gender, education, occupation, marital status for example, details in Supplement Table S5). Two studies adjusted for the impact of neighbourhood self-selection in the form of neighbourhood preference [46,47].

**Table 1:** Summary of included studies and their characteristics.

Characteristic	Attribute (number of studies)
Study Design	Cross-Sectional (*n* = 50); Longitudinal (*n* = 1)
Location	Brazil (*n* = 28); Chile (n = 1); Colombia (*n* = 11); Mexico (*n* = 12); Singapore (n = 8)
Age Group	Adult (*n* = 33); Children/Adolescents (*n* = 9); Adult & Children/Adolescents (*n* = 7)
Age range (years)	6–98
Unit of Analysis	Individual (*n* = 49); Community (n = 2)
PA Outcome	Recreational walking (n = 12); Transportation walking/biking (*n* = 25); General cycling/biking (n = 3); Moderate to vigorous PA (n = 11); Leisure-time PA (n = 6); Total PA (*n* = 2);
PA Measure Type	Self-reported (*n* = 40); Objective (n = 7); Both (*n* = 4)
BE Feature Type	Residential/Population Density (n = 32); Street Connectivity (*n* = 32); Mixed Land Use (n = 32); Retail Floor (n = 3); Public Space/Parks/Open Space (*n* = 31); Recreation land use proximity (n = 12); Non-recreational land use proximity (n = 8); Transit stops presence/proximity/access (*n* = 19); Trails/sidewalks/pathways/cycleways (*n* = 15); Street lighting/shade (*n* = 6); Walkability/Pedestrian index (*n* = 4) Urban sprawl/Urban form (n = 1); Other (*n* = 5)

Note: BE – Built Environment; PA- Physical Activity.

Single studies may include multiple attributes.

### 3.2 Measurement of built environment

Desktop Mapping (80%) was the most common method used to measure the BE, followed by field auditing (13%), Global Positioning System (GPS) (5%) and remote sensing (2%). Of the BE features present in the included studies, residential density (housing or people density), street connectivity and mixed land use were most frequently reported (63%), followed by presence of green spaces (alternatives called public spaces, parks, open spaces) (61%).

Other features were reported less commonly: proximity or access to public transport (37%), availability of paths for walking or cycling (29%), proximity to spaces for recreation (24%), proximity to non-recreational land uses (e.g. food stores and gyms) (16%), presence of pedestrian amenities such as street lighting and features providing shade (12%), a derived single measure of walkability (8%), and area designated for retail (6%).

After categorizing BE measurements using the Walkability for Health framework, the number of studies and BE-PA relationships by BE feature are presented in Table 2. The most reported BE feature related to PA was land use mix (34 relationships, 16 studies). Additional BE features reported were primarily intersection density (32 relationships, 21 studies), presence and proximity of transit stops (25 relationships, 8 studies), and residential density (23 relationships, 11 studies). Only one study highlighted the presence of wheelchair access and police surveillance, while no studies examined the presence or proximity of parking features.

**Table 2:** Built environment characterization using the walkability for health framework in SIDS and LA.

Neighbourhood Design Domain	BE Feature	Number of Studies	Number of Relationships
Surveillance	Presence of Street Lighting	4	6
	Police Surveillance	1	1
Experience	Presence of Trees	2	4
	Clean environment^a^	4	7
	Sidewalk maintenance	3	4
	Road Maintenance^b^	3	4
	Pedestrian infrastructure and facilities	5	7
	Slope	5	5
Parking	Non-street parking	0	0
	Distance to parking facilities	0	0
Traffic Safety	Bike path/ Lane	9	13
	Bus stop/ transit station	8	25
	Sidewalk presence	3	3
	Road Signage^c^	2	5
	Wheelchair Access	1	2
	Traffic Lights/Buffer	3	4
Community	Public Open Spaces	6	13
	Presence of pedestrian comfort facilities^d^	3	3
	Proximity to Open Spaces	2	2
Greenspace	Presence of Parks	15	17
	Presence of Trees	2	4
	Presence of open greenspace	3	3
	Proximity to Greenspace	4	4
Density	Residential Density^e^	11	23
	Dwelling/Housing Density^f^	3	4
	Population Density	8	13
Connectivity	Intersection Density	21	32
	Street Density	6	10
	Street length	4	7
Land Use	Land Use Mix^g^	16	34
	Presence of recreational places	7	13
	Presence of activity places	4	9
	Retail Land Use	3	5
	Civic Land Use	2	4
	Presence of Health Facilities	6	9

Note: ^a^Absence of litter and poor building maintenance; sewage maintaince; garbage maintenance.

^b^Restoration of road surface for commuting without any unsafe interruptions.

^c^Stop signs; traffic direction signs; crossing signs.

^d^Presence of outdoor seating or benches.

^e^Total number of dwelling housing units in areas designated for residential usage.

^f^Number of dwelling/housing units with statistical administrative units.

^g^Estimated using five main land use types: residential, retail, entertainment, office and institutional combined into a single index known as the entropy index.

### 3.3 Measurement of physical activity

A total of 40 studies measured physical activity by self-report, with 11 reporting objective measures. Types of physical activity most frequently reported was active commuting (transportation walking/cycling/biking) (*n* = 29 studies), followed by moderate to vigorous PA (*n* = 15 studies), recreational walking (*n* = 12 studies), leisurely activity (e.g., any form of physical activity; active park use; sport participation and frequency of outdoor play) (*n* = 11 studies), general walking for any purpose (*n* = 8 studies), and general cycling and biking (*n* = 5 studies).

### 3.4 Evidence gap matrix

**Fig. 2:**
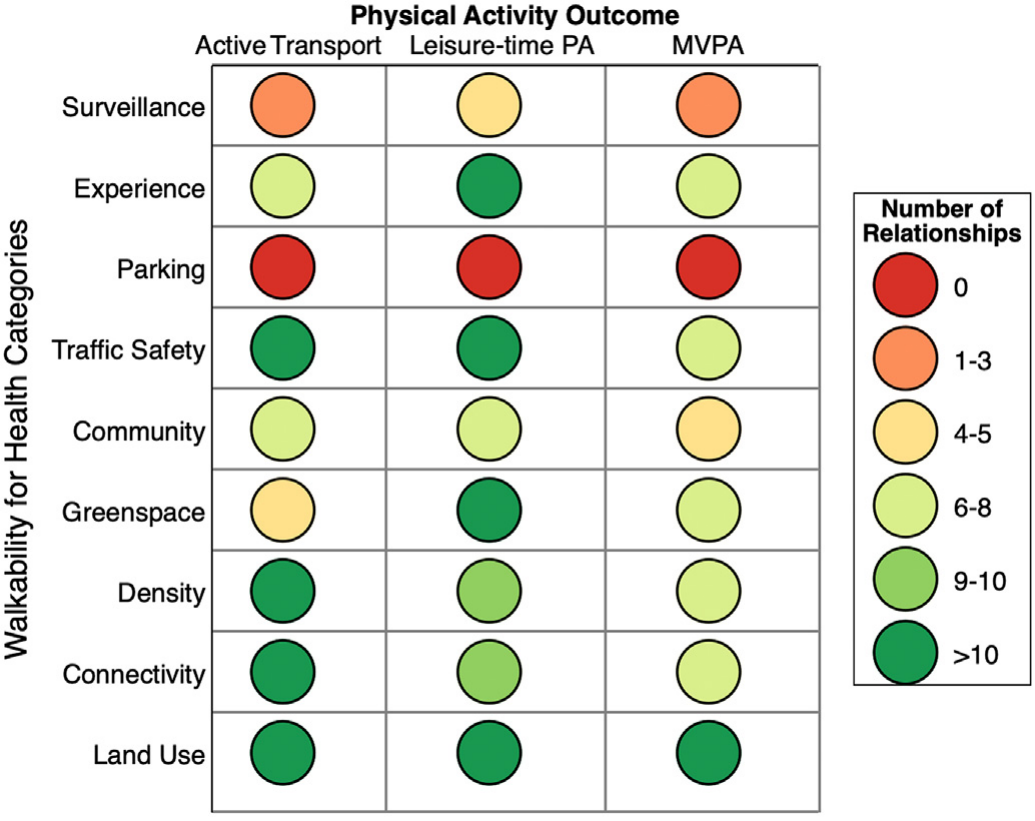
Evidence gap matrix of number of relationships, stratified by BE domains and PA outcomes.

There were 306 relationships between BE measures and PA outcomes. An evidence gap-map of these relationships, stratified by Walkability for Health domains and by physical activity type is presented in Fig. 2. Across all BE and PA domains, land use was the most commonly reported (82 relationships), followed by traffic safety, connectivity and density. Whereas the least commonly reported BE domain across all PA outcomes was parking (0 relationships), surveillance (7 relationships), and community (19 relationships).

### 3.5 Relationships between the built environment and physical activity

**Fig. 3:**
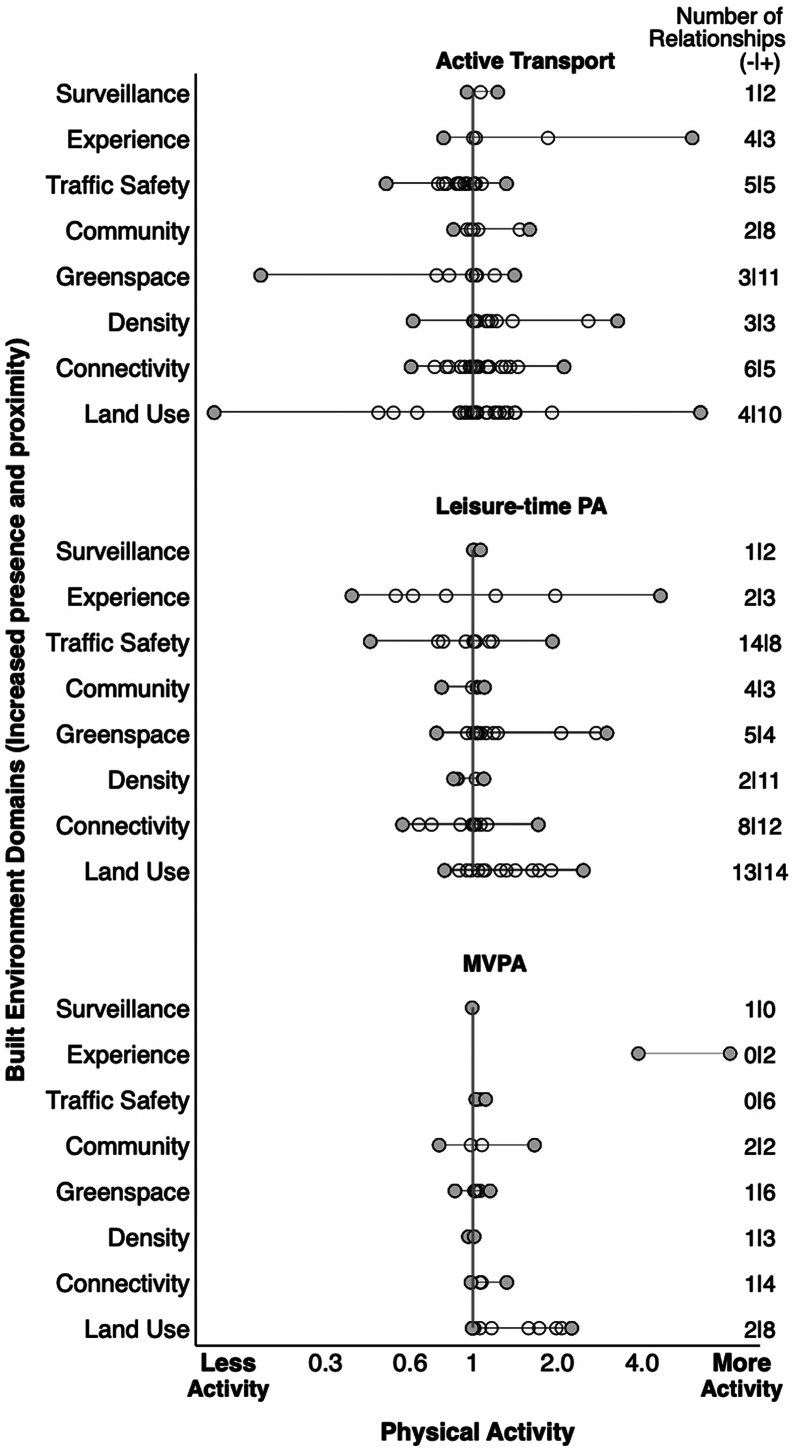
Equiplot plot of relationships between PA outcomes and BE domains.

The direction of the relationships between PA outcomes and BE domains are described in Fig. 3. There were 220 relationships with adequate confounder adjusted odds ratio information which ranged from 0.12–8.17. Overall, 61% (*n* = 135) of relationships were positive (OR > 1.00) while 39% (*n* = 85) were negative (OR ≤ 1.00). For active transport we found 106 relationships with BE domains. Higher residential density had the highest difference of positive compared to negative relationships (11 positive | 2 negative) followed by increased street connectivity (12 positive | 8 negative) when linked with increased active commuting. We observed that traffic safety had the highest difference of negative to positive relationships (8 positive | 14 negative). For leisure-time PA we found 75 relationships with BE domains. Increased presence and proximity of outdoor greenspaces had the highest difference of positive compared to negative relationships (11 positive | 3 negative) followed by land use (10 positive | 4 negative) and community (8 positive | 2 negative) when linked with increased leisure-time PA. We observed that experience (increased presence of features linked to outdoor comfort) (3 positive | 4 negative) and increased street connectivity (5 positive | 6 negative) had the highest difference of negative to positive relationships. For MVPA, we found 39 relationships with BE domains. Increased presence of traffic calming, and safety features had the highest difference of positive compared to negative relationships (6 positive | 0 negative). This was followed by land use (8 positive | 2 negative) and greenspace (6 positive | 1 negative).

### 3.6 Publication bias assessment

**Fig. 4:**
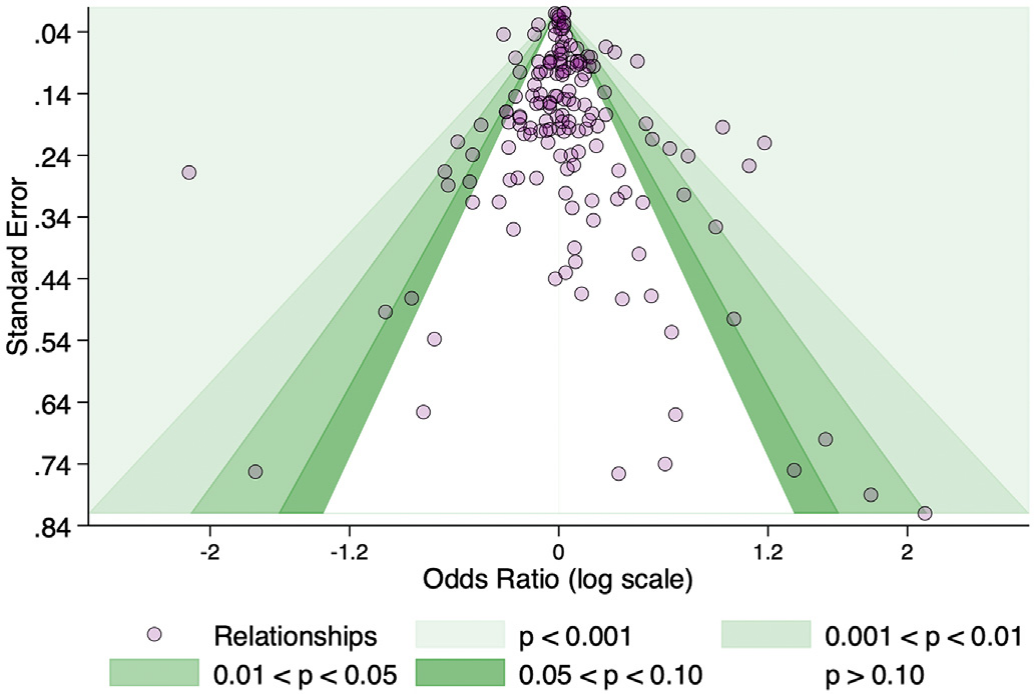
Contour-enhanced funnel plot of relationships between PA outcomes and BE characteristics.

Fig. 4 presents a contour-enhanced funnel plot of relationships between BE features and PA outcomes. Studies from the IPEN (International Physical Activity Network) study [5,[48], [49], [50], [51]] were excluded from the present analysis because only aggregated estimates were reported which included estimates from settings of North American; Europe and Oceania. From visual inspection of the plot there was no clear evidence of asymmetry. This was further examined using Egger test which showed no evidence of publication bias (Bias = 0.14, 95% CI -0.20 to 0.49, *p* = 0.411). We found similar results by PA category [See supplement Fig. S1].

## 4 Discussion

### 4.1 Overview of results

Our scoping review sought to summarize and characterize the evidence highlighting the influence of BE characteristics on PA in settings located in SIDS and LA. We found 51 articles describing 306 BE-PA relationships. Latin America had most of the evidence primarily in Brazil, Chile, Colombia, and Mexico, while Singapore was the only SIDS setting with available evidence. Our review showed that there is a critical lack of evidence from the Caribbean region.

### 4.2 Can the Caribbean use evidence from other SIDS?

In the absence of Caribbean studies, evidence from other SIDS is potentially applicable due to their shared environmental characteristics. However, we found a similar evidence gap in non-Caribbean SIDS, with Singapore the only country for which peer-reviewed evidence on BE-PA relationships exists. Whether Singapore is sufficiently similar to Caribbean SIDS for parallels to be drawn is debatable. There are some parallels between Singapore and the Caribbean, mainly related to their scarce land resources and high population density in urban areas [52,53]. In addition, they have similar World Bank economic classifications, with Singapore defined as high income and most Caribbean states as high or upper middle income (World Bank, 2021). However, the similar World Bank classifications disguise considerable differences, and by most economic metrics, Singapore is fundamentally different from the Caribbean. For example, the average per capita income in 2020 in Caribbean SIDS was US$8900, compared with US$59,800 in Singapore [54]. In 2020, Singapore was ranked globally as the largest per capita exporter of goods and services with a monetary per capita value of US$49,500, whereas Trinidad and Tobago, the largest per capita exporter in the Caribbean, was ranked at 32 with a monetary value of US$4500 [55]. This economic advantage, combined with the necessity of planned redevelopment following the Second World War, makes the built environment of Singapore unique among the SIDS. Since 1971, Singapore's urban planning process has been guided by an overarching framework, which was developed with input from the United Nations, as well as the economic, social, environmental and infrastructure development sectors in Singapore. The framework focuses not only on economic performance but also considers quality of life of residents in urban sectors [56,57]. This contrasts with the largely unplanned urban development seen in the Caribbean and limits the utility of evidence from Singapore for understanding BE-PA relationships in the Caribbean.

### 4.3 Is evidence from LA useful for the Caribbean?

The majority of the articles included in this review were based on research conducted in LA, mainly Brazil, Chile, Colombia, and Mexico. These countries are geographically close to the Caribbean, but are culturally distinct and have different urban structures due to their large land areas. Brazil, for example, has a land mass of 8.5 million square kilometres [58]. In contrast, SIDS are characterized by their small size, with seven out of the fifteen Caribbean SIDS having land areas smaller than 800 km^2^ [59]. This impacts urban form, as a single urban centre may be accessed by the entire island, albeit not always by walking. Research on environmental influences on physical activity has focused on the concept of “walkable neighbourhoods”, defined by the ease with which people can walk or cycle from place to place. It is unclear whether this concept applies to small islands, where the places people live, work, and spend their leisure time are often spread across the entire country rather than being concentrated in neighbourhoods. It is possible that the BE features known to encourage walking do so universally: the findings of the IPEN study demonstrate that when comparable objective measures of the BE are used, the relationship between the BE and physical activity is generally similar across diverse cities [48]. However, until the role of the BE in promoting physical activity has been studied in SIDS, we are unable to determine the extent to which these relationships are influenced by the developmental and cultural differences that characterize their environments.

### 4.4 Summary of BE-PA relationships

Many areas of the BE have been identified to aid in increasing individual and community physical levels which has universal benefit to improving overall health and well-being. We found that features related to increasing residential and retail density and active transport most commonly improved levels of physical activity. Evidence from developed settings have shown that neighbourhoods of higher residential density may lead to increased daily active commuting and leisurely activity among residents [13,60]. This may be due to the fact that areas of higher residential and population density may have a more diverse land use mix and be more interconnected [61]. However, issues related to safety should not be ignored when applied to ageing populations found in the Caribbean.

Our review found that most BE-PA relationships were associated with land use BE features. Presence and proximity to commercial destinations and areas with a mixture of designated commercial, residential and educational land uses were associated with increased PA across all domains. This finding is consistent with evidence produced in North American and European settings [24,62]. Presence and proximity to commercial and recreation land use areas within communities can help support the promotion of increased active commuting and leisurely activity among residents. This potentially can lead to more opportunities for increased interpersonal social interaction [63] however this may not be consistent across all settings [64]. Additionally, we found that spaces for social interaction and open greenspaces showed the most positive compared to negative relationships within leisure-time PA. Public open spaces have been identified as an environmental support for leisurely activities that aids in promoting physical and psychological health and social well-being. The rapid growth of urban areas in the Caribbean may have led to a reduction in the quantity or size of these social spaces [65]. Keeping public open spaces within neighbourhoods is important to aid in increasing residential social capital by acting as a meeting place for users, allowing for the development and maintenance of social bonds [66].

Moreover, we also found that increased presence and proximity to open greenspaces such as parks had the most positive compared negative relationships within MVPA. Studies in non-LA-SIDS settings have highlight the lack of association between presence of greenspaces and MVPA (Chong et al., 2019). Participation in outdoor fitness activities has transitioned to indoor gyms thus usage of outdoor greenspace has been decreasing especially in the tropical region of the Caribbean. Similar to public open spaces, greenspaces provides opportunities to increase social capital within communities by acting as a meeting place for users, allowing for the development and maintenance of social bonds [66]. These greenspaces are attractive areas for engaging in PA based on their scenic and outdoor appeal, and cleanliness [67].

From an urban planning point of view in the Caribbean, consideration of open greenspaces has been of interest to encourage outdoor recreational PA through establishment of parks in urban areas. Built environment and physical published evidence in the Caribbean is limited however, a study conducted by Cunningham-Myrie and colleagues examined the use of a well-known park for physical activity in Jamaica. They noted that park usage was primarily linked to evening time use by persons 18–64 years predominated by females. Proximity to the central business district, safety and aesthetic appeal were the main attractors for its use [68]. Although these findings are specific to one Caribbean country, they can be applied to other countries within the region who are faced with similar structural urban environments. Urban design policy makers within the region should consider evidence built environment and PA evidence emerging from the region to incorporate the findings from this review and other regionally relevant studies into built environment intervention design targeted at motivating an increase in outdoor physical activity across children, adults and the elderly to help combat the burden of NCDs in the region.

### 4.5 Strengths and limitations

To the best of our knowledge, this is the first study attempting to characterize and link the BE using objective measures and relate its association with physical activity with a goal of applying it to Caribbean region. Our review has limitations. Firstly, in keeping with most scoping reviews, we did not systematically assess bias inherent in the included studies. Secondly, our review did not include grey literature, which may have reduced the number of reported BE features in relation to PA. Thirdly, the decision to include non-randomized evidence contributed to the heterogeneity of methods used to objectively measure BE features, which affects the comparability of findings obtained. Lastly, most studies did not measure nor adjust for neighbourhood self-selection which is known to be an important confounder in attenuating BE-PA relationships [[69], [70], [71], [72], [73]].

### 4.6 Implications for practice

• The most frequently reported built environment features related to increased physical activity land use, greenspace and density.
• Findings from this review may provide evidence for the need for future multidisciplinary regional and international collaboration for the design of built environment studies in the Caribbean.
• There is no definitive evidence on which BE features are linked with promoting PA in the Caribbean; however these findings can help support BE professionals regionally and internationally when considering the Caribbean BE.
• Although the review suggests BE avenues for promoting active commuting and leisurely PA, translational research is needed to understand whether results can be successfully adapted for SIDS, which have unusual urban structures, often a single urban centre with connections to outlying communities.

## 5 Conclusion

This is the first review of its kind that attempts to synthesize available evidence related to the BE and PA giving us a better understanding of the contextual issues faced in the Caribbean region. We can conclude that there are some aspects of the BE which were shown to encourage more PA in settings similar to the Caribbean. Our findings highlight the opportunity of designing local neighbourhoods to have a mix of land uses, high connectivity and residential density, and availability open greenspaces to encourage higher levels of PA within neighbourhoods. The combination of changes to the BE with other health promotion and behaviour change interventions may help reduce the burden of physical inactivity in the Caribbean. Stronger BE-PA relationships should become more apparent as the number of studies continues to grow over time with more robust research designs.

## Author contributions

Conceptualization: KR, CH & IH. Data curation: KR & CH. Formal analysis: KR, CH & IH. Writing - original draft, review & editing: KR, CH & IH. All authors have read and agreed to the published version of the manuscript.

## Funding

This review is not funded by any research grant or external funding agency.

## Compliance with ethical standards

The present study does not include human nor animal subjects, therefore institutional ethical approval was not required.

## Declaration of Competing Interest

The authors declare that they have no conflict of interests.

## Appendix A Supplementary data

Supplementary material Image 1
