# Supplementary material for: Understanding the relationship between built environment features and physical activity in the Caribbean: A scoping review
Source: Dialogues Health. 2022 Dec 5;2:100088. doi: 10.1016/j.dialog.2022.100088 (PMC10953899; doi:10.1016/j.dialog.2022.100088)
Supplement: Supplementary file 1 — Supplementary material [file mmc1.docx]

Table S1: Preferred Reporting Items for Systematic reviews and Meta-Analyses extension for Scoping Reviews (PRISMA-ScR) Checklist

| **SECTION** | **ITEM** | **PRISMA-ScR CHECKLIST ITEM** | **REPORTED ON PAGE #** |
| --- | --- | --- | --- |
| **TITLE** | | | |
| Title | 1 | Identify the report as a scoping review. | 1 |
| **ABSTRACT** | | | |
| Structured summary | 2 | Provide a structured summary that includes (as applicable): background, objectives, eligibility criteria, sources of evidence, charting methods, results, and conclusions that relate to the review questions and objectives. | 1-2 |
| **INTRODUCTION** | | | |
| Rationale | 3 | Describe the rationale for the review in the context of what is already known. Explain why the review questions/objectives lend themselves to a scoping review approach. | 3-4 |
| Objectives | 4 | Provide an explicit statement of the questions and objectives being addressed with reference to their key elements (e.g., population or participants, concepts, and context) or other relevant key elements used to conceptualize the review questions and/or objectives. | 4 |
| **METHODS** | | | |
| Protocol and registration | 5 | Indicate whether a review protocol exists; state if and where it can be accessed (e.g., a Web address); and if available, provide registration information, including the registration number. | 4-5 |
| Eligibility criteria | 6 | Specify characteristics of the sources of evidence used as eligibility criteria (e.g., years considered, language, and publication status), and provide a rationale. | 5 |
| Information sources* | 7 | Describe all information sources in the search (e.g., databases with dates of coverage and contact with authors to identify additional sources), as well as the date the most recent search was executed. | 5 |
| Search | 8 | Present the full electronic search strategy for at least 1 database, including any limits used, such that it could be repeated. | 5-6 |
| Selection of sources of evidence† | 9 | State the process for selecting sources of evidence (i.e., screening and eligibility) included in the scoping review. | 5-6 |
| Data charting process‡ | 10 | Describe the methods of charting data from the included sources of evidence (e.g., calibrated forms or forms that have been tested by the team before their use, and whether data charting was done independently or in duplicate) and any processes for obtaining and confirming data from investigators. | 5-6 |
| Data items | 11 | List and define all variables for which data were sought and any assumptions and simplifications made. | 6 |
| Critical appraisal of individual sources of evidence§ | 12 | If done, provide a rationale for conducting a critical appraisal of included sources of evidence; describe the methods used and how this information was used in any data synthesis (if appropriate). | 7 |
| Synthesis of results | 13 | Describe the methods of handling and summarizing the data that were charted. | 7 |
| **RESULTS** | | | |
| Selection of sources of evidence | 14 | Give numbers of sources of evidence screened, assessed for eligibility, and included in the review, with reasons for exclusions at each stage, ideally using a flow diagram. | 7-8 |
| Characteristics of sources of evidence | 15 | For each source of evidence, present characteristics for which data were charted and provide the citations. | 9-10 |
| Critical appraisal within sources of evidence | 16 | If done, present data on critical appraisal of included sources of evidence (see item 12). | 9 |
| Results of individual sources of evidence | 17 | For each included source of evidence, present the relevant data that were charted that relate to the review questions and objectives. | 10-13 |
| Synthesis of results | 18 | Summarize and/or present the charting results as they relate to the review questions and objectives. | 13-17 |
| **DISCUSSION** | | | |
| Summary of evidence | 19 | Summarize the main results (including an overview of concepts, themes, and types of evidence available), link to the review questions and objectives, and consider the relevance to key groups. | 18, 20-21 |
| Limitations | 20 | Discuss the limitations of the scoping review process. | 21 |
| Conclusions | 21 | Provide a general interpretation of the results with respect to the review questions and objectives, as well as potential implications and/or next steps. | 22 |
| **FUNDING** | | | |
| Funding | 22 | Describe sources of funding for the included sources of evidence, as well as sources of funding for the scoping review. Describe the role of the funders of the scoping review. | 23 |

JBI = Joanna Briggs Institute; PRISMA-ScR = Preferred Reporting Items for Systematic reviews and Meta-Analyses extension for Scoping Reviews.

* Where *sources of evidence* (see second footnote) are compiled from, such as bibliographic databases, social media platforms, and Web sites.

† A more inclusive/heterogeneous term used to account for the different types of evidence or data sources (e.g., quantitative and/or qualitative research, expert opinion, and policy documents) that may be eligible in a scoping review as opposed to only studies. This is not to be confused with *information sources* (see first footnote).

‡ The frameworks by Arksey and O’Malley (6) and Levac and colleagues (7) and the JBI guidance (4, 5) refer to the process of data extraction in a scoping review as data charting*.*

§ The process of systematically examining research evidence to assess its validity, results, and relevance before using it to inform a decision. This term is used for items 12 and 19 instead of "risk of bias" (which is more applicable to systematic reviews of interventions) to include and acknowledge the various sources of evidence that may be used in a scoping review (e.g., quantitative and/or qualitative research, expert opinion, and policy document).

*From:* Tricco AC, Lillie E, Zarin W, O'Brien KK, Colquhoun H, Levac D, et al. PRISMA Extension for Scoping Reviews (PRISMAScR): Checklist and Explanation. Ann Intern Med. 2018;169:467–473. [doi: 10.7326/M18-0850](http://annals.org/aim/fullarticle/2700389/prisma-extension-scoping-reviews-prisma-scr-checklist-explanation).

**Search Terms**

***Built environment terms***

Neighbourhood OR neighborhood OR spatial OR environment OR GIS OR “geographic information systems” OR “built environment” OR “physical environment” OR “urban form” OR “street scape” OR “streetscape” OR “walkability” OR “macroscale walkability” OR “urban planning” OR “pedestrian friendly” OR “walkable” OR cycleable OR “cyclability” OR “land use” OR “residential density” OR “mixed use” OR “mixed land use” OR “mixed land-use” OR “retail floor area” OR “retail floor” OR “street connectivity” OR “road connectivity” OR “road intersections” OR “street intersections” OR “bus stop” OR “bus stops” OR “bus stop density” OR “public transport” OR “transport” OR “urban design” OR “open space” OR “green space” OR “parks” OR “street layout” OR pathway OR “street pathway” OR “cycle paths” OR “urban walkability” OR “urban sprawl” OR “spatial” OR “park” OR “sidewalk” OR “urban” OR “suburban” OR “bike-friendly” OR “bike friendly” OR “active design”

***Physical Activity Terms***

“Physical Activity” OR exercise OR inactivity OR “physical exercise” OR “physical inactivity” OR “phys*” OR bicycling OR strolling OR “leisure time” OR sports OR recreation OR “recreation activity” OR “active transport” OR pedestrian OR “pedestrian activity” OR “active travel” OR “active living” OR “active recreation” OR walk OR liveable OR “cycl*” OR cycle OR cycle OR “healthy lifestyle” OR sedentary OR “sedentary lifestyle” OR “sedentary activity” OR “moderate activity” OR “vigorous activity” OR “objective physical activity” OR “pedometers” OR steps OR calories OR “mode share” OR “active learning”

***SIDS (Small Island Developing States) Terms***

“small island developing state”[TIAB] OR “small island developing states”[TIAB] OR pacific islands OR caribbean region OR west indies OR  anguilla[TIAB] OR “american samoa” [TIAB] OR antigua[TIAB] OR barbuda[TIAB] OR aruba[TIAB] OR bahamas[TIAB] OR bahrain[TIAB] OR barbados[TIAB] OR belize[TIAB] OR “british virgin islands” [TIAB] OR “cape verde” [TIAB] OR “cook islands” [TIAB] OR cuba[TIAB] OR dominica[TIAB] OR “dominican republic” [TIAB] OR “federated states of micronesia” [TIAB] OR fiji[TIAB] OR “french polynesia” [TIAB] OR grenada[TIAB] OR guam[TIAB] OR guinea-bissau[TIAB] OR guyana[TIAB] OR haiti[TIAB] OR jamaica[TIAB] OR kiribati[TIAB] OR maldives[TIAB] OR “marshall islands” [TIAB] OR mauritius[TIAB] OR montserrat[TIAB] OR nauru[TIAB] OR “netherlands antilles” [TIAB] OR “new caledonia” [TIAB] OR niue[TIAB] OR “northern mariana islands” [TIAB] OR palau[TIAB] OR “papua new guinea” [TIAB] OR “puerto rico” [TIAB] OR “st. kitts” [TIAB] OR “saint kitts” [TIAB] OR “nevis” [TIAB] OR “saint kitts and nevis” [TIAB] OR “st. lucia” [TIAB] OR “saint lucia” [TIAB] OR “saint vincent” [TIAB] OR “st. vincent” [TIAB] OR grenadines[TIAB] OR “st. vincent and the grenadines” [TIAB] OR “saint vincent and the grenadines” [TIAB] OR samoa[TIAB] OR “sao tome” [TIAB] OR principe[TIAB] OR “sao tome and principe” [TIAB] OR seychelles[TIAB] OR singapore[TIAB] OR “solomon islands” [TIAB] OR suriname[TIAB] OR timor-leste[TIAB] OR tonga[TIAB] OR trinidad[TIAB] OR tobago[TIAB] OR “trinidad and tobago” [TIAB] OR tuvalu[TIAB] OR “united states virgin islands” [TIAB] OR vanuatu[TIAB]

***Latin American Countries Terms***

 “latin america”[TIAB] OR “latin americas”[TIAB] OR brazil[TIAB] OR mexico[TIAB] OR colombia[TIAB] OR argentina[TIAB] OR peru[TIAB] OR venezuela[TIAB] OR chile[TIAB] OR guatemala[TIAB] OR ecuador[TIAB] OR bolivia[TIAB] OR honduras[TIAB] OR paraguay[TIAB] OR nicaragua[TIAB] OR “el salvador” [TIAB] OR “costa rica” [TIAB] OR panama[TIAB] OR uruguay[TIAB] OR guadeloupe[TIAB]

***Additional Caribbean Countries Terms***

martinique[TIAB] OR “french guiana” [TIAB] OR “saint martin” [TIAB] OR “st. martin” [TIAB] OR “saint barthelemy” [TIAB] OR “st. barthelemy” [TIAB]

Table S2: List of Small Isand Developing States and Latin America

| **UN Group** | **Geographic Region** | | |
| --- | --- | --- | --- |
|  | **AIMS** | **Caribbean** | **Pacific** |
| UN Member | Bahrain, Cabo Verde, Comoros, Guinea-Bissau, Maldives, Mauritius, Sao Tome and Principe, Seychelles, Singapore | Antigua and Barbuda, Bahamas, Barbados, Belize, Cuba, Dominica, Dominican Republic, Grenada, Guyana, Haiti, Jamaica, Saint Kitts and Nevis, Saint Lucia, Saint Vincent and the Grenadines, Suriname, Trinidad and Tobago | Fiji, Kiribati, Marshall Islands, Micronesia, Nauru, Palau, Papua New Guinea, Samoa, Solomon Islands, Timor-Leste, Tonga, Tuvalu, Vanuatu |
| Non-UN Member |  | Anguilla, Aruba, Bermuda, British Virgin Islands, Cayman Islands, Curacao, Guadeloupe, Martinique, Montserrat, Puerto Rico, Sint Maarten, Turks and Caicos Islands, US Virgin Islands | American Samoa, Commonwealth of Northern Marianas, Cook Islands, French Polynesia, Guam, New Caledonia, Niue |

Table S3: List of Countries with Latin America and the Caribbean

| **Group** | **Geographic Region** | | |
| --- | --- | --- | --- |
|  | **North and Central America** | **Caribbean** | **South America** |
| Spanish-speaking | Costa Rica, El Salvador, Guatemala, Honduras, Mexico, Nicaragua, Panama | Cuba, Dominican Republic, Puerto Rico | Argentina, Bolivia, Chile, Colombia, Ecuador, Paraguay, Peru, Uruguay, Venezuela |
| Non-Spanish-Non-English-speaking |  | Curacao, Haiti, Guadeloupe, Martinique, Saint-Barthelemy, Sint Maarten | Brazil, French Guiana, Suriname |
| English-speaking | Belize | Antigua and Barbuda, Bahamas, Barbados, Belize, Anguilla, Aruba, Bermuda, British Virgin Islands, Cayman Islands, Dominica, Grenada, Guyana, Jamaica, Saint Kitts and Nevis, Saint Lucia, Saint Vincent and the Grenadines, Suriname, Trinidad and Tobago, Turks and Caicos Islands, US Virgin Islands | Guyana |

Table S4: Walkability for Health Framework neighbourhood design domains categorizing the walkability of the built environment (1).

| **Neighbourhood Design** | **Summary** | **Examples** |
| --- | --- | --- |
| **Surveillance** | Outdoor infrastructure design allowing individuals to observe pedestrians in the street from different viewpoints and presence of neighbourhood patrolling | - Street lighting - Location of buildings and their entrances near streets - Building includes balconies and porches overlooking the street - Patrolling of law enforcement within the community |
| **Experience** | Neighbourhood design provides a pleasant area for a physical activity experience in relation to the streetscape, aesthetics, thermal comfort, slope and way finding. | - Maintenance neighbourhood infrastructure sidewalks - Inclusion of trees and vegetation along streets, water bodies, landmarks, high fences and signage. |
| **Parking** | Reduced parking availability and presence of parking away from streets segments | - Parking location behind buildings or in basements with entries towards secondary streets - Parking in off street locations |
| **Traffic Safety** | Allowance of pedestrians primary preference over cars in streets. Providing safe and comfortable access to bus stops and reliable bus service. Slowdown of traffic due to traffic calming treatments | - Providing ample sidewalks and bike lanes - Provide frequent and reliable bus service that connects to the rest of the city - Bus stops have accompanied with it a safe and covered sitting area - Sidewalks and bike lanes buffer (stripe of vegetation) present and on street parking - Implementation of traffic calming treatments (speed limit signage, speed bumps, pedestrian only streets) |
| **Community** | Provision of spaces for social interactions within the neighbourhood | - Inclusion of civic space, community centre, shared facilities and spaces for community gathering - Encouragement of neighbourhood participation in organizations |
| **Greenspace** | Inclusion of varieties of greenspace in size and proximity with ease of access | - Homes within nearby proximity to viewing the outdoor greenspace - Inclusion of greenspace 1/5/10 minute(s) walking distance from home |
| **Density** | Neighbourhoods must have high residential and retail density maintaining the pedestrian scale at the street level | - Inclusion of different types of housing options within the neighbourhood |
| **Connectivity** | Providing street networks that gives multiple and short routes | - Inclusion of grid street network - Inclusion of multiple 4-way intersections - Exclusion of dead end streets |
| **Land-use** | Mixture of land uses so that there is multiple destinations for walking/commuting | - Location of services within a comfortable commuting distance from home - Presence of areas with food stores, schools, recreational facilities |

Table S5**:** Summary of included studies

| **Author, year** | **Country & Population Type** | **Cross-Sectional or Longitudinal data collection** | **Sample size** | **Unit of analysis** | **Built Environment Features measured** | **Physical Activity Type** | **Confounders** | **Main Study findings** |
| --- | --- | --- | --- | --- | --- | --- | --- | --- |
| Andrade, 2019 (2) | Brazil,  Adult | Cross-sectional | 3815 | Individual | Mixed Land-Use;  Public Space/Parks/Open Space; Trails/sidewalks/path/cycle ways;  Pedestrian amenities | Recreational walking  (Self-reported) | age, sex, current occupation, marital status, time of residence | Individuals living in census tracts with higher walking environment indicators and safety were more likely to be active during leisure time |
| Bautista-Hernandez, 2021 (3) | Mexico,  Adult/Children/Adolescents | Cross-sectional | 64355 | Individual | Residential Density; Street Connectivity; Mixed Land-Use; Transit proximity/access | Transportation walking; General cycling/biking  (Self-reported) | Gender, age, kin, educational attainment, household socioeconomic status, availability of biking infrastructure | Factors associated with bike use were distance to the centre, density of mass-transit systems stations, street intersection density, and the flat surface. |
| Bojorquez, 2018 (4) | Mexico,  Adult | Cross-sectional | 2345 | Individual | Public Space/Parks/Open Space; Recreational land use proximity | Recreational walking, Moderate to vigorous physical activity  (Self-reported) | age, body mass index, diabetes, hypertension, occupation, children, marital status, socioeconomic level, education, most frequently used transport | No interaction between access to public spaces and public spaces quality in their effect on physical activity. There was an association between the presence of public spaces in the 400m buffer, and higher odds of being in the low physical activity level |
| Borjorquez, 2021 (5) | Mexico,  Adult | Cross-sectional | 2928 | Individual | Mixed Land-Use; Public Space/Parks/Open Space; Slope; Pedestrian facilities | Transportation walking; Leisure-time PA; Total PA  (Self-reported) | age, socioeconomic level, years of education and occupation | No quantitative association between access to public spaces and physical activity was found, as well as no interactions between access to public spaces and other variables. |
| Borchardt, 2019 (6) | Brazil,  Adult | Cross-sectional | 1300 | Individual | Residential/Population Density; Public Space/Parks/Open Space; Recreation land use proximity; Transit proximity/access; Pedestrian amenities;  Proximity to sea front,  proportion of households without nearby open sewage,  proportion of households with manhole/down take pipes | Recreational walking; Transportation walking; Moderate to vigorous physical activity  (Self-reported) | gender, age, skin colour, marital status; assets index, dwelling time in neighbourhood | Few associations between environmental variables and PA practice were identified. Only proximity to the seafront, presence of private gyms or sports clubs and higher average monthly income of the household head were associated with the practice of PA. |
| Cerin, 2017 (7) | Mexico  Brazil  Colombia;  Adult | Cross-sectional | 6712 | Individual | Residential/Population Density; Street Connectivity;  Mixed Land-Use;  Retail Floor;  Public Space/Parks/Open Space; Recreation land use proximity; Non-recreational land use proximity;  Transit proximity/access | Moderate to vigorous physical activity  (Objectively measured) | age, gender, education, employment status and marital status | Time of the day, day of week, gender and employment status were significant moderators of environment- MVPA associations. |
| Cerin, 2018 (8) | Mexico  Brazil  Colombia;  Adult | Cross-sectional | 6712 | Individual | Residential/Population Density; Street Connectivity; Mixed Land-Use; Retail Floor; Public Space/Parks/Open Space; Recreation land use proximity; Non-recreational land use proximity;  Transit proximity/access | Moderate to vigorous physical activity  (Objectively measured) | Age, sex, educational attainment, work status, neighbourhood SES | Objective net residential density, public transport density, and number of parks in the neighbourhood were consistently associated with MVPA |
| Chen, 2020 (9) | Singapore  Children/  Adolescents | Cross-sectional | 73 | Individual | Pedestrian amenities (street lighting/shade/furniture) | Moderate to Vigorous PA  (Objective & Self-reported) | age, sex, ethnicity, body status, main caregiver, parental education level, marital status, housing | MVPA was similar throughout the week, and SB was slightly higher on non-school days. In preschools, SOPLAY showed more children engaging in MVPA outdoors than indoors, and absence of portable active play equipment. |
| Christiansen, 2016 (10) | Mexico  Brazil  Colombia;  Adult | Cross-sectional | 11674 | Individual | Residential/Population Density; Street Connectivity;  Mixed Land-Use;  Public Space/Parks/Open Space | Transportation walking; General cycling/biking  (Self-reported) | age, sex, marital status, educational attainment, employment status | Positive associations of walking and cycling for transport with all the environmental features |
| Custódio, 2021 (11) | Brazil,  Adult | Cross-sectional | 2591 | Individual | Mixed Land-Use | Moderate to Vigorous PA  (Objective) | Sex and geographic location | Women and the people who use POS during the week or in the morning are more likely to practice MVPA. |
| Da Silva, 2017 (12) | Brazil,  Children/  Adolescents | Cross-sectional | 5249 | Individual | Residential/Population Density; Street Connectivity;  Public Space/Parks/Open Space; Recreation land use proximity; Trails/sidewalks/pathways/ cycle ways;  Pedestrian amenities | Recreational & General walking; MVPA; Active Transport  (Self-Reported) | sex, time living at that address and socioeconomic status | Street lighting was positively associated with objectively-measured MVPA and proportion of paved streets and buffer's average family income were associated with lower MVPA. Living near the beach increased the odds of leisure-time MVPA activity. |
| Dias, 2019 (13) | Brazil,  Children/  Adolescents | Cross-sectional | 1130 | Individual | Residential/Population Density; Street Connectivity;  Public Space/Parks/Open Space; Recreation land use proximity; Trails/sidewalks/pathways/ cycle ways;  density of blocks, average size of the blocks | Transportation walking or cycling to school  (Self-reported) | perceived environmental factors, sex, age, socioeconomic status and class | Neighbourhood recreation facilities, as well as objectively measured existence of bicycle paths and residential density in 0.5 km and 1 km buffers around adolescents' home. Existence of parks and squares was also associated with ACS |
| Dias, 2020 (14) | Brazil,  Children/  Adolescents | Cross-sectional | 1113 | Individual | Residential/Population Density; Street Connectivity; Public Space/Parks/Open Space; Walkability/pedestrian index | Leisure-time PA  (Self-reported) | age, geographic region, perceived environment | Leisure walking was positively associated with access to services and lower distance to parks and squares in girls. Residential density were associated with leisure walking in girls. Neighbourhood recreation facilities was positively associated with leisure walking. Land use mix, neighbourhood recreation facilities and places for walking were positively associated with leisure walking |
| Dias, 2021 (15) | Brazil,  Children/  Adolescents | Cross-sectional | 1123 | Individual | Residential/Population Density; Street Connectivity | Transportation walking  (Self-reported) | sex, age, socioeconomic status | Residential density is a mediator on the association between ACS and perceived environmental factors, including land-use mix diversity, neighbourhood recreation facilities , and access to services. Connectivity between streets did not correlate with ACS |
| Faerstein, 2018 (16) | Brazil,  Adult | Longitudinal | 1731 | Individual | Street Connectivity;  Public Space/Parks/Open Space; Recreation land use proximity; Non-recreational land use proximity; Trails/sidewalks/path/cycle ways | Any form of physical activity  (Self-reported) | Sex, age, skin colour/race, education, family income per capita in monthly minimum wages, marital status | Compared to participants living in the upper quartile of distance to waterfronts, those living in its lowest quartile had 2.6-fold higher odds of reporting non-work PA in all 4 study waves. |
| Florindo, 2019 (17) | Brazil,  Adult | Cross-sectional | 3145 | Individual | Mixed Land Use;  Public Space/Parks/Open Space; Non-recreational land use proximity;  Transit proximity/access; Trails/sidewalks/pathways/ cycle ways | Transportation walking  (Self-reported) | age, sex, education, marital status, obesity status based on BMI, smoking ; self-reported health; employment situation; car or motorcycle ownership; length of living in the same residence; family per capita income; health administration areas ; and safety perception for physical activity | The presence of public transportation stations, destinations mix, was significantly associated with walking for transportation for 150 min or more per week. |
| Giehl, 2016 (18) | Brazil,  Adult | Cross-sectional | 12911 | Individual | Residential/Population Density; Street Connectivity;  Mixed Land-Use;  Public Space/Parks Trails/sidewalks/path/cycle ways; Pedestrian amenities | Recreational walking; Transportation walking; General walking  (Self-reported) | gender, age, education | Individuals living in neighbourhoods with a higher population density, with a higher street connectivity, a higher sidewalk proportion, and paved streets were more likely to walk for transportation. |
| Gomes, 2016 (19) | Brazil,  Adult | Cross-sectional | 5779 | Individual | Residential/Population Density; Public Space/Parks/Open Space; Recreation land use proximity | Recreational walking; General walking  (Self-reported) | education, gender, age, marital status, skin colour, consumption recommended of fruits or vegetables (FV) (five or more per day for five or more days of the week), consumption of meat with visible fat, consumption of sweetened beverages five or more days per week, smoking, and perceived poor health status. | After adjusting for individual characteristics, the increase of density of private places for physical activity and the smaller homicide rate in the neighbourhood increased physical activity in leisure time. |
| Gomes, 2021 (20) | Brazil,  Adult | Cross-sectional | 5779 | Individual | Residential/Population Density; Mixed Land-Use Public Space/Parks/Open Space | Leisure-time PA  (Self-reported) | sex, age, education level and martial status | There was a higher density of places for LTPA practice, higher population and residential density, and higher family income in the cluster. |
| Gomez, 2010 (21) | Colombia,  Adult | Cross-sectional | 1966 | Individual | Street Connectivity;  Mixed Land Use;  Public Space/Parks/Open Space; Recreation land use proximity; Transit proximity/access | Recreational walking; General walking  (Self-reported) | gender, age groups, education level, limitation to engage in physical activity, and SES. | People who lived in areas with middle park area were more likely to walk for at least 60 minutes during a usual week. Participants who reported feeling safe or very safe from traffic were more likely to report walking for at least 60 minutes. |
| Gomez, 2010a (22) | Colombia,  Adult | Cross-sectional | 1315 | Individual | Residential/Population Density; Mixed Land-Use;  Public Space/Parks/Open Space; Transit proximity/access; Trails/sidewalks/path/cycle ways | Recreational walking/Leisure time physical activity  (Self-reported) | gender, age group, education level | Compared with inactive people, those who resided in neighbourhoods with the highest tertile dedicated to parks were more likely to be regularly active. Those who resided in neighbourhoods with presence of (mass public transportation system) were more likely to be irregularly active as compared with inactive people. |
| Gonzalez, 2020 (23) | Colombia,  Children/  Adolescents | Cross-sectional | 2845 | Individual | Non-recreational land use | Transportation walking  (Self-reported) | age, sex, parental education, number of motorised vehicles and crime perception score | Greater distance to school and vehicle ownership were associated with a lower likelihood of engaging in AST (active school transport) in sites in upper-middle- and high-income countries. Crime perception was negatively associated to AST only in sites in high-income countries. |
| Guerra, 2018 (24) | Mexico,  Adult/City | Cross-sectional | 2.46 Million | Community | Residential/Population Density; Street Connectivity; Sprawl/Urban sprawl/Urban form | Transportation walking; Commuting biking  (Self-reported) | Gender, age, education, income, job population imbalance | Commuters were also less likely to drive in areas with better public transit supply. Collectively the measures of urban form were as strongly related to the probability someone commutes to work by car as household income. |
| Guerra, 2021 (25) | Mexico,  Adult | Cross-sectional | 2435133 | Individual | Residential/Population Density; Street Connectivity | Transportation walking  (Self-reported) | Gender, age, household income, household size, vehicle availability, occupation, highest educational attainment, housing type, job-area imbalance | In USA and Mexico, urban residents living in housing types associated with more centrally located housing in more densely populated urban areas with less roadway are less likely to commute by private vehicle than similar residents in other housing types and other urban areas. |
| Guzman, 2020 (26) | Colombia,  Adult/  Children/  Adolescents | Cross-sectional | 21378 | Individual | Residential/Population Density; Street Connectivity; Mixed Land-use; Public Space/Parks/Open Space | Transportation walking  (Self-reported) | Age, gender, occupation, SES, car ownership, education level | People living in areas were the land-use mix and job/population ratio are low, tend to have longer walking distances, suggesting the need to implement special policies that reallocate some activities and encourage higher land-use mix in the urban periphery where low-income households tend to be located. |
| Higuera-Mendieta, 2021 (27) | Colombia,  Adult/  Adolescents | Cross-sectional | 16495 | Individual | Street Connectivity; Mixed Land-use; Transit proximity/access; Trails/sidewalks/pathways/ cycle ways | Bicycle commuting | Occupation, driver's license status, household SES, crime, number of collisions, age, commute distance | Among women, the availability of bike paths at the trip destination was positively associated with bicycling, while age and being a student were negatively associated with bicycling. Living in areas with the lowest socio-economic status was positively associated with bicycling, while having a driver's license and living close to bus rapid transit stations were negatively associated with bicycling. |
| Hino, 2011(28) | Brazil,  Adult Children/  Adolescents | Cross-sectional | 1206 | Individual | Residential/Population Density; Public Space/Parks/Open Space; Recreation land use proximity; Density of recreational facilities | Recreational walking  (Self-reported) | sex, age, education, marital status, skin colour, car ownership, BMI | Walking during leisure time was associated with area income level, having≥2 gyms vs. none and distance to recreation centres.  MVPA was associated with neighbourhood income and having ≥2 gyms vs. none. |
| Hino, 2012 (29) | Brazil,  Adult | Cross-sectional | 699 | Individual | Residential/Population Density; Street Connectivity;  Mixed Land-Use | Moderate to vigorous physical activity (Objective measured) | Income | 16 high-level sectors were included walkability and 16 below walkability, eight low-income and eight high-income in each category. |
| Hino, 2013 (30) | Brazil,  Adult | Cross-sectional | 2097 | Individual | Residential/Population Density; Street Connectivity;  Mixed Land-Use;  Recreation land use proximity; Non-recreational land use proximity | Transportation walking  (Self-reported) | sex, age, education, marital status, car ownership and BMI | Higher density of Bus Rapid Transit stations, and the proportion of residential and commercial areas were associated with any walking prevalence. Higher access to bike paths was inversely associated with walking at recommended levels. Greater number of traffic lights, and higher land use mix were inversely associated with cycling. |
| Hino, 2019 (31) | Brazil,  Adult | Cross-sectional | 699 | Individual | Public Space/Parks/Open Space | Moderate to vigorous physical activity  (Self-reported) | sex, age group, education, time at work/school, children at home, satisfaction with neighbourhood | The amount of public leisure spaces, within a radius of 500 meters, with one or more equipment for PA was negatively associated with the practice of walking. The number of public leisure spaces within a radius of 1,000 meters was positively associated with moderate to vigorous physical activities. The proximity and the amount of public leisure spaces are associated with higher levels of moderate to vigorous PA in adults. |
| Hou, 2019 (32) | Singapore,  Adult | Cross-sectional | 25922 | Community | Residential/Population Density; Street Connectivity;  Mixed Land-Use;  Public Space/Parks/Open Space, Transit proximity/access; Trails/sidewalks/path/cycle ways | Transportation walking  (Self-reported) | age, gender, license ownership, ethnicity, employment status, household characteristics, neighbourhood socioeconomic context; weather conditions | Better access to different tiers of planned urban centres is associated with more walking trips for some age groups of older people but less walking trips for other age groups. Neighbourhood-level land use characteristics such as higher density and better street connectivity significantly increase walking trips for all adults with no differential effects across age. |
| Hou, 2020 (33) | Singapore,  Adult | Cross-sectional | 900 | Individual | Residential/Population Density; Street Connectivity;  Public Space/Parks/Open Space, Transit proximity/access; Pedestrian amenities | Transportation walking  (Self-reported) | age, gender, education, occupation, income, housing socioeconomic status | Perceived access to recreational facilities exert independent positive effects daily walking trip frequency and enhance the effects of comparable objective measures The results also find positive effects of perceptions of transit proximity and comfortable and safe pedestrian connections on older adults' daily transit trip-making. |
| Larranaga, 2016 (34) | Brazil,  Adult  Children/  Adolescents | Cross-sectional | 442 | Individual | Residential/Population Density; Street Connectivity;  Mixed Land-Use;  Transit proximity/access | Transportation & General walking  (Self-reported) | age, gender, education level, car availability, income level | The results of the elasticities computed for the number of walking trips with respect to the built environment variables were smaller than in other studies. Only the effect of population density on walking frequency seems to be marginally higher |
| Lee, 2016 (35) | Mexico,  Children/  Adolescents | Cross-sectional | 1321 | Individual | Residential/Population Density; Street Connectivity;  Mixed Land-Use; Tails/sidewalks/path/cycle ways | Frequency of outdoor play (Self-reported) | child age, gender, annual household income, and the number of children and the total number of people in the household | Fewer path obstructions and more pedestrian amenities were associated positively with outdoor play. Greater street cleanliness, more pedestrian amenities, and more path obstructions were associated with less participation in sports or organized activities. Walkability was negatively associated with all physical activities. |
| Lim, 2017 (36) | Singapore,  Adult | Cross-sectional | 1972 | Individual | Mixed Land Use; Public Space/Parks/Open Space;  Transit proximity/access | General walking & Participation in sports  (Self-reported) | Gender, Age group, Marital status, Employment status, Ethnic group, Housing type, diet, medical history | Increased distance (geographical inaccessibility) to a train station was significantly associated with lower odds of participation in sports whereas greater distance to a subsidized private clinic was associated with lower odds of having high cholesterol diagnosed. |
| Lopes, 2018 (37) | Brazil,  Adult | Cross-sectional | 1419 | Individual | Street Connectivity;  Mixed Land Use;  Transit proximity/access | Transportation walking; Cycling for transportation  (Self-reported) | Gender, age. marital status, socioeconomic status, nutritional status. self-rated health and quality of life, perceived crime, number of motor vehicles in household | Medium "streetscape" score was inversely associated with walking ≥ 150min/week and bicycling. |
| Mello, 2020 (38) | Brazil,  Children/  Adolescents | Cross-sectional | 236 | Individual | Residential/Population Density; Street Connectivity;  Mixed Land-Use; ­Non-recreational land use; Walkability/pedestrian index | Transportation walking; General walking  (Self-reported) | gender, age | The commuting to school was associated with residence distance to school and walkability. The gender moderates the relationship between walkability and commuting to school, association only in girls. The relationship between the use of public spaces and PA was reduced in the presence of cardiorespiratory fitness |
| Mo, 2018 (39) | Singapore,  Adult | Cross-sectional | 10000 | Individual | Residential/Population Density; Mixed Land-Use;  Transit proximity/access | Transportation walking  (Self-reported) | type of residence, citizenship, number of people in household, children under 6, age, gender, driver’s license, employment status, income, commute trip | The built environment-especially distance to MRT station, transportation infrastructures, land-use mix, and socioeconomic activities- significantly influences the first- and last-mile travel behaviours. |
| Nakamura, 2016 (40) | Brazil,  Adult | Cross-sectional | 1588 | Individual | Residential/Population Density; Mixed Land-Use | Recreational walking, Moderate to vigorous physical activity  (Self-reported) | Sex, age, marital status, educational level, number of cars per home, BMI | Walking during leisure-time was negatively associated with population density higher than 68 km/m2 . Moderate intensity physical activity was not associated with built environment factors. There were different associations between the built environment factors with leisure time PA except for moderate intensity physical activity. |
| Neves, 2021 (41) | Brazil,  Adult | Cross-sectional | 15775 | Individual | Residential/Population Density; Street Connectivity;  Mixed Land-Use; Transit proximity/access | Transportation walking  (Self-reported) | Age, gender, education, employment, family income | For the city of Sao Paulo, built environment variables are more relevant at the origin and the dimension most related to walking choice is diversity, probably due to socio- economic reason. |
| Nyunt, 2015 (42) | Singapore,  Adult | Cross-sectional | 402 | Individual | Residential/Population Density; Street Connectivity;  Mixed Land-Use;  Public Space/Parks/Open Space | Transportation walking  (Self-reported) | Gender, Age, Ethnicity, Education, Housing status, self-rated health status, POMA Balance score, POMA Gait score, perceived walkability | Objective GIS measure of Accessibility Index have positively significant independent associations with transportation physical activity, after adjusting for demographics, socio-economic and health status. |
| Oliva, 2018 (43) | Chile,  Adult | Cross-sectional | 1487 | Individual | Residential/Population Density; Street Connectivity;  Mixed Land Use;  Retail Floor;  Public Space/Parks/Open Space; Transit proximity/access; Trails/sidewalk/path/cycle ways | Cycling for transport  (Self-reported) | Car at home, Bicycles at home, Student vs employed, income, gender, household size | It was found that, at the origin, residential density and bike lanes length have a positive effect on cycling commuting. |
| Parra, 2010 (44) | Colombia,  Adult | Cross-sectional | 1966 | Individual | Street Connectivity;  Mixed Land Use;  Public Space/Parks/Open Space | Frequency of active park use  (Self-reported) | Age, gender, education level, slope of the terrain | Residents from areas with higher park density and high land use mix were more likely to report active park use, while those from areas of high connectivity were less likely |
| Pentrunoff, 2021 (45) | Singapore,  Adult | Cross-sectional | 3435 | Individual | Public Space/Parks/Open Space | Leisure-time PA  (Self-reported) | age, gender, martial status, household income, education, BMI | Better perceived but not true park access was significantly associated with greater park use. Park access (perceived or true) was not associated with physical activity time in parks. Greater participant park time and physical activity time in parks were associated with higher wellbeing scores |
| Rosas-Satizabal, 2020 (46) | Colombia,  Adult  Children/  Adolescents | Cross-sectional | 968 | Individual | Non-recreational land use | General cycling/biking  (Self-reported) | Age, gender, job role, education level, driver's license status | Marked differences in potential accessibility to work and study opportunities between and within clusters. |
| Sallis, 2016 (47) | Mexico/  Brazil/  Colombia;  Adult | Cross-sectional | 14222 | Individual | Residential/Population Density; Street Connectivity;  Mixed Land-Use;  Public Space/Parks/Open Space, Transit proximity/access; Trails/sidewalks/path/cycle ways | Moderate to vigorous physical activity  (Objective measured) | age, sex, education, marital status , employment status, city, accelerometer wear time, and SES of administrative unit | Four of six environmental features were significantly, positively, and linearly related to physical activity. Mixed land use and distance to nearest public transport point were not related to physical activity. |
| Salvo, 2014 (48) | Mexico,  Adult | Cross-sectional | 662 | Individual | Residential/Population Density; Street Connectivity;  Mixed Land-Use;  Public Space/Parks/Open Space, Transit proximity/access; Walkability/pedestrian index | Moderate to vigorous physical activity  (Objective measured) | Age, sex, education, marital status, individual-level SES, motor vehicle ownership, BMI | Walkability was inversely related to total weekly minutes of MVPA and weekly minutes of MVPA within bouts. |
| Schipperijn, 2017 (49) | Mexico &  Brazil;  Adult | Cross-sectional | 6181 | Individual | Public Space/Parks/Open Space | Recreational walking & Moderate to vigorous physical activity  (Both self-reported and objective measured) | age, gender, education, marital status, employment status, city, accelerometer wear time, and administrative-unit-level SES | More parks within 1 km from participants' homes were associated with greater leisure-time physical activity and accelerometer-measured MVPA. |
| Silva, 2020 (50) | Brazil,  Adult  Children/  Adolescents | Cross-sectional | 493 | Individual | Trails/sidewalk/pathways/ cycle ways; Pedestrian amenities | Transportation walking  (Self-reported) | Gender, age, SES, vehicle ownership, type of school administration | Presence of safety signs was inversely associated with active commuting. Traffic safety and distance to school were associated with active commuting to school among the study participants |
| Siqueirra, 2013 (51) | Brazil,  Adult | Cross-sectional | 697 | Individual | Residential/Population Density; Mixed Land-Use | Recreational walking; Transportation walking; Moderate to vigorous physical activity  (Self-reported) | age; gender; of education; marital status; children in the household; number of cars in household; and time living in the neighbourhood | Walkability showed an independent association with walking for transport and leisure-time MVPA. |
| Song, 2020 (52) | Singapore,  Adult | Cross-sectional | 810 | Individual | Public Space/Parks/Open Space; Transit proximity/access; Trails/sidewalk/pathways/ cycle ways; | Recreational walking; Transportation walking  (Self-reported) | Age, gender, ethnicity, education, work status, occupational role, work role, household income, type of dwelling, smoking, drinking, eating behaviour | The accessibility to destinations and other walk- ability features could promote transportation or recreational outdoor physical activity and therefore, health among older residents in Singapore. Interestingly, built environment characteristics related differently to physical activity undertaken for transportation and recreational purposes. |

Figure S1: Contour-enhanced funnel plot of BE-PA Relationships by PA outcome

References

1. Zuniga Teran AA. From Neighborhoods to Wellbeing and Conservation: Enhancing the Use of Greenspace through Walkability [unpublished thesis]. [Arizona]: University of Arizona; 2015.

2. Andrade A, Mingoti S, Costa D, Xavier C, Proietti F, Caiaffa W, et al. Built and Social Environment by Systematic Social Observation and Leisure-Time Physical Activity Report among Brazilian Adults: a Population-Based Study. JOURNAL OF URBAN HEALTH-BULLETIN OF THE NEW YORK ACADEMY OF MEDICINE. 2019;96(5):682–91.

3. Bautista-Hernández DA. Mode choice in commuting and the built environment in México City. Is there a chance for non-motorized travel? Journal of Transport Geography. 2021;92:103024.

4. Bojorquez I, Ojeda-Revah L, Diaz R. Access to public spaces and physical activity for Mexican adult women. Cadernos de saude publica. 2018;34:e00065217.

5. Bojorquez I, Romo-Aguilar M de L, Ojeda-Revah L, Tena F, Lara-Valencia F, García H, et al. Public spaces and physical activity in adults: insights from a mixed-methods study. Cad Saúde Pública. 2021;37(1).

6. Borchardt JL, Paulitsch RG, Dumith SC. The influence of built, natural and social environment on physical activity among adults and elderly in southern Brazil: a population-based study. International journal of public health. 2019;64(5):649–58.

7. Cerin E, Mitáš J, Cain KL, Conway TL, Adams MA, Schofield G, et al. Do associations between objectively-assessed physical activity and neighbourhood environment attributes vary by time of the day and day of the week? IPEN adult study. International Journal of Behavioral Nutrition and Physical Activity. 2017;14(1):34.

8. Cerin E, Conway TL, Adams MA, Barnett A, Cain KL, Owen N, et al. Objectively-assessed neighbourhood destination accessibility and physical activity in adults from 10 countries: an analysis of moderators and perceptions as mediators. Social Science & Medicine. 2018;211:282–93.

9. Chen B, Waters CN, Compier T, Uijtdewilligen L, Petrunoff NA, Lim YW, et al. Understanding physical activity and sedentary behaviour among preschool-aged children in Singapore: a mixed-methods approach. BMJ Open. 2020;10(4):e030606.

10. Christiansen LB, Cerin E, Badland H, Kerr J, Davey R, Troelsen J, et al. International comparisons of the associations between objective measures of the built environment and transport-related walking and cycling: IPEN adult study. Journal of Transport & Health. 2016;3(4):467–78.

11. Custódio IG, Lopes AA dos S, Kopp D, Silva AT da, de Chaves RN, Rodriguez-Añez CR, et al. Padrão de utilização de espaços públicos abertos e nível de atividade física em São José dos Pinhais, Paraná. Rev Bras Ciênc Esporte. 2021;43.

12. da Silva ICM, Hino AA, Lopes A, Ekelund U, Brage S, Gonçalves H, et al. Built environment and physical activity: domain-and activity-specific associations among Brazilian adolescents. BMC Public Health. 2017;17(1):616.

13. Dias AF, Gaya AR, Pizarro AN, Brand C, Mendes TM, Mota J, et al. Perceived and objective measures of neighborhood environment: Association with active commuting to school by socioeconomic status in Brazilian adolescents. Journal of Transport & Health. 2019;14:100612.

14. Dias AF, Gaya AR, Santos MP, Brand C, Pizarro AN, Fochesatto CF, et al. Neighborhood environmental factors associated with leisure walking in adolescents. Rev Saúde Pública. 2020;54.

15. Dias AF, Gaya AR, Brand C, Florindo AA, Villa-González E, García-Hermoso A, et al. Mediation role of residential density on the association between perceived environmental factors and active commuting to school in Brazilian adolescents. Cad Saude Publica. 2021;37(5):e00067620.

16. Faerstein E, da Silveira IH, Boclin K de LS, Curioni CC, de Castro IRR, Junger WL. Associations of neighborhood socioeconomic, natural and built environmental characteristics with a 13-year trajectory of non-work physical activity among civil servants in Rio de Janeiro, Brazil: The Pro-Saude Study. Health & place. 2018;53:110–6.

17. Florindo AA, Barbosa JP dos AS, Barrozo LV, Andrade DR, de Aguiar BS, Failla MA, et al. Walking for transportation and built environment in Sao Paulo city, Brazil. Journal of Transport & Health. 2019;15:100611.

18. Giehl MWC, Hallal PC, Corseuil CW, Schneider IJC, d’Orsi E. Built environment and walking behavior among Brazilian older adults: a population-based study. Journal of physical activity and health. 2016;13(6):617–24.

19. Gomes CS, Matozinhos FP, Mendes LL, Pessoa MC, Velasquez-Melendez G. Physical and social environment are associated to leisure time physical activity in adults of a brazilian city: a cross-sectional study. PLoS One. 2016;11(2):e0150017.

20. Gomes CS, Vieira CS, Rocha FL, Temponi HR, Vieira MAS, Mendes MS, et al. Spatial analysis of leisure-time physical activity in an urban area. Rev bras epidemiol. 2021;24:e210012–e210012.

21. Gomez LF, Sarmiento OL, Parra DC, Schmid TL, Pratt M, Jacoby E, et al. Characteristics of the built environment associated with leisure-time physical activity among adults in Bogotá, Colombia: a multilevel study. Journal of physical activity & health. 2010 Jul;7:S196-203.

22. Gomez L, Parra D, Buchner D, Brownson R, Sarmiento O, Pinzon J, et al. Built Environment Attributes and Walking Patterns Among the Elderly Population in Bogota. AMERICAN JOURNAL OF PREVENTIVE MEDICINE. 2010;38(6):592–9.

23. González SA, Sarmiento OL, Lemoine PD, Larouche R, Meisel JD, Tremblay MS, et al. Active School Transport among Children from Canada, Colombia, Finland, South Africa, and the United States: A Tale of Two Journeys. Int J Environ Res Public Health [Internet]. 2020 [cited 5AD Jan 1];17(11). Available from: https://pubmed.ncbi.nlm.nih.gov/32481728/

24. Guerra E, Caudillo C, Monkkonen P, Montejano J. Urban form, transit supply, and travel behavior in Latin America: Evidence from Mexico’s 100 largest urban areas. Transport Policy. 2018;69:98–105.

25. Guerra E, Li M. The relationship between urban form and mode choice in US and Mexican cities: A comparative analysis of workers’ commutes. Journal of Transport and Land Use. 2021;14(1):pp 441-462.

26. Guzman LA, Peña J, Carrasco JA. Assessing the role of the built environment and sociodemographic characteristics on walking travel distances in Bogotá. Journal of Transport Geography [Internet]. 2020 [cited 10AD Jan 1];88. Available from: ["http://www.sciencedirect.com/science/article/pii/S0966692319310786", "https://trid.trb.org/view/1737924"]

27. Higuera-Mendieta D, Uriza PA, Cabrales SA, Medaglia AL, Guzman LA, Sarmiento OL. Is the built-environment at origin, on route, and at destination associated with bicycle commuting? A gender-informed approach. J Transp Geogr. 2021;94:None.

28. Hino AA, Reis RS, Sarmiento OL, Parra DC, Brownson RC. The built environment and recreational physical activity among adults in Curitiba, Brazil. Preventive medicine. 2011;52(6):419–22.

29. Hino AA, Rech CR, Goncalves PB, Hallal PC, Reis RS. Projeto ESPAÇOS de Curitiba, Brazil: applicability of mixed research methods and geo-referenced information in studies about physical activity and built environments. Revista panamericana de salud publica= Pan American journal of public health. 2012;32(3):226–33.

30. Hoehner CM, Ribeiro IC, Parra DC, Reis RS, Azevedo MR, Hino AA, et al. Physical activity interventions in Latin America: expanding and classifying the evidence. Am J Prev Med. 2013 Mar;44(3):e31-40.

31. Hino AAF, Rech CR, Gonçalves PB, Reis RS. Accessibility to public spaces for leisure and physical activity in adults in Curitiba, Paraná State, Brazil. Cadernos de Saúde Pública. 2019;35(12).

32. Hou Y. Polycentric urban form and non-work travel in Singapore: A focus on seniors. Transportation Research Part D: Transport and Environment. 2019;73:245–75.

33. Hou Y, Yap W, Chua R, Song S, Yuen B. The associations between older adults’ daily travel pattern and objective and perceived built environment: A study of three neighbourhoods in Singapore. Transport Policy. 2020;99:pp 314-328.

34. Larranaga A, Rizzi L, Arellana J, Strambi O, Cybis H, Larranaga AM, et al. The influence of built environment and travel attitudes on walking: A case study of Porto Alegre, Brazil. INTERNATIONAL JOURNAL OF SUSTAINABLE TRANSPORTATION. 2016;10(4):332–42.

35. Lee RE, Soltero EG, Jáuregui A, Mama SK, Barquera S, Jauregui E, et al. Disentangling Associations of Neighborhood Street Scale Elements With Physical Activity in Mexican School Children. Environment and Behavior. 2016 Jan 1;48(1):150–71.

36. Lim KK, Kwan YH, Tan CS, Low LL, Chua AP, Lee WY, et al. The association between distance to public amenities and cardiovascular risk factors among lower income Singaporeans. Preventive Medicine Reports. 2017 Dec 1;8:116–21.

37. Lopes AA dos S, Kienteka M, Fermino RC, Reis RS. Characteristics of the environmental microscale and walking and bicycling for transportation among adults in Curitiba, Paraná State, Brazil. Cadernos de Saúde Pública [Internet]. 2018 [cited 2020 Sep 21];34(1). Available from: http://www.scielo.br/scielo.php?script=sci_abstract&pid=S0102-311X2018000105014&lng=en&nrm=iso&tlng=en

38. Mello JB, Duncan M, Dias A, Bergmann G, Gaya AR, Gaya A. Biological and urban environmental variables as correlates of adolescents’ physical activity. Rev bras ativ fís saúde. 2020;25:1–10.

39. Mo B, Shen Y, Zhao J. Impact of Built Environment on First- and Last-Mile Travel Mode Choice: Transportation Research Record [Internet]. 2018 Jul 31 [cited 2020 Sep 21]; Available from: https://journals.sagepub.com/doi/10.1177/0361198118788423

40. Nakamura PM, Teixeira IP, Hino AAF, Kerr J, Kokubun E, Nakamura PM, et al. Association between private and public places and practice of physical activity in adults. Revista Brasileira de Cineantropometria &amp; Desempenho Humano. 2016 Jun;18(3):297–310.

41. Neves CET, da Silva AR, de Arruda FS. Exploring the link between built environment and walking choice in São Paulo city, Brazil. Journal of Transport Geography [Internet]. 2021 [cited 5AD Jan 1];93. Available from: ["http://www.sciencedirect.com/science/article/pii/S0966692321001174", "https://trid.trb.org/view/1849400"]

42. Nyunt MSZ, Shuvo FK, Eng JY, Yap KB, Scherer S, Hee LM, et al. Objective and subjective measures of neighborhood environment (NE): relationships with transportation physical activity among older persons. International Journal of Behavioral Nutrition and Physical Activity. 2015 Sep 15;12(1):108.

43. Oliva I, Galilea P, Hurtubia R. Identifying cycling-inducing neighborhoods: A latent class approach. International Journal of Sustainable Transportation. 2018 Nov 26;12(10):701–13.

44. Parra DC, Gomez LF, Fleischer NL, David Pinzon J. Built environment characteristics and perceived active park use among older adults: results from a multilevel study in Bogotá. Health Place. 2010 Nov;16(6):1174–81.

45. Petrunoff NA, Yi NX, Dickens B, Sia A, Koo J, Cook AR, et al. Associations of park access, park use and physical activity in parks with wellbeing in an Asian urban environment: a cross-sectional study. Int J Behav Nutr Phys Act. 2021;18(1):87.

46. Rosas-Satizábal D, Guzman LA, Oviedo D. Cycling diversity, accessibility, and equality: An analysis of cycling commuting in Bogotá. Transportation Research Part D: Transport and Environment [Internet]. 2020 [cited 11AD Jan 1];88. Available from: ["http://www.sciencedirect.com/science/article/pii/S1361920920307495", "https://trid.trb.org/view/1742224"]

47. Sallis JF, Cerin E, Conway TL, Adams MA, Frank LD, Pratt M, et al. Physical activity in relation to urban environments in 14 cities worldwide: a cross-sectional study. Lancet. 2016 May 28;387(10034):2207–17.

48. Salvo D, Reis RS, Stein AD, Rivera J, Martorell R, Pratt M. Characteristics of the Built Environment in Relation to Objectively Measured Physical Activity Among Mexican Adults, 2011. Prev Chronic Dis [Internet]. 2014 Aug 28 [cited 2019 Aug 5];11. Available from: https://www.ncbi.nlm.nih.gov/pmc/articles/PMC4149324/

49. Schipperijn J, Cerin E, Adams MA, Reis R, Smith G, Cain K, et al. Access to parks and physical activity: an eight country comparison. Urban For Urban Green. 2017 Oct;27:253–63.

50. Silva AAP, Lopes AADS, Silva JSB, Prado CV, Reis RS. Characteristics of the schools’ surrounding environment, distance from home and active commuting in adolescents from Curitiba, Brazil. Rev Bras Epidemiol. 2020;23:e200065.

51. Siqueira Reis R, Hino AAF, Ricardo Rech C, Kerr J, Curi Hallal P. Walkability and physical activity: findings from Curitiba, Brazil. American journal of preventive medicine. 2013 Sep;45(3):269–75.

52. Song S, Yap W, Hou Y, Yuen B. Neighbourhood Built Environment, Physical Activity, and Physical Health among Older Adults in Singapore: A Simultaneous Equations Approach. Journal of Transport & Health [Internet]. 2020 [cited 9AD Jan 1];18. Available from: ["http://www.sciencedirect.com/science/article/pii/S2214140520300852", "https://trid.trb.org/view/1714726"]
